# Altered EEG variability on different time scales in participants with autism spectrum disorder: an exploratory study

**DOI:** 10.1038/s41598-022-17304-x

**Published:** 2022-07-29

**Authors:** Lukas Hecker, Mareike Wilson, Ludger Tebartz van Elst, Jürgen Kornmeier

**Affiliations:** 1 Department of Psychiatry and Psychotherapy, University of Freiburg Medical Center, Freiburg, Germany; 2 Faculty of Medicine, University of Freiburg, Freiburg, Germany; 3 Faculty of Biology, University of Freiburg, Freiburg, Germany; 4 Perception and Cognition Lab, Institute for Frontier Areas of Psychology and Mental Health (IGPP), Freiburg, Germany; 5 Department of Psychosomatic Medicine and Psychotherapy, University of Freiburg Medical Center, Freiburg, Germany

**Keywords:** Developmental biology, Neuroscience, Biomarkers

## Abstract

One of the great challenges in psychiatry is finding reliable biomarkers that may allow for more accurate diagnosis and treatment of patients. Neural variability received increasing attention in recent years as a potential biomarker. In the present explorative study we investigated temporal variability in visually evoked EEG activity in a cohort of 16 adult participants with Asperger Syndrome (AS) and 19 neurotypical (NT) controls. Participants performed a visual oddball task using fine and coarse checkerboard stimuli. We investigated various measures of neural variability and found effects on multiple time scales. (1) As opposed to the previous studies, we found reduced inter-trial variability in the AS group compared to NT. (2) This effect builds up over the entire course of a 5-min experiment and (3) seems to be based on smaller variability of neural background activity in AS compared to NTs. The here reported variability effects come with considerably large effect sizes, making them promising candidates for potentially reliable biomarkers in psychiatric diagnostics. The observed pattern of universality across different time scales and stimulation conditions indicates trait-like effects. Further research with a new and larger set of participants are thus needed to verify or falsify our findings.

## Introduction

### Autism spectrum disorder (“ASD”)

The autism spectrum disorder (ASD) describes a developmental condition characterized by abnormal function in reciprocal social interaction, communication and restricted, stereotyped, repetitive behavior^1^.

Clinical diagnostic is so far mainly based on behavioral variables and developmental histories^2^. As a consequence, the diagnostic work strongly depends on the professional experience of the examiners. Moreover, the pattern of behavioral symptoms can change over lifetime (e.g.,^2^). More objective physiological markers are yet missing but urgently necessary. Studies on physiological markers exist, but the reported findings are controversial (e.g.,^2,3^).

### Altered visual processing in ASD

Altered lower-level sensory processing has only recently been shown to provide potentially interesting markers in ASD (e.g.,^2,4^) and has now been integrated in the DSM-V^5^. Individuals with ASD are often oversensitive to loud noises or bright colors. Others, in contrast, are attracted to light and fascinated by reflections and bright-colored objects (e.g.,^6^).

Perceptual interpretations of individuals with ASD are often dominated by small sensory details, whereas they have difficulties to integrate spatial context^7^ and prior perceptual experiences (e.g.,^8–12^). Further, many ASD observers are less susceptible to visual illusions (e.g.,^2,13,14^). Basic retinal visual functions in ASD have been shown to be normal^15^ pointing to higher levels of visual pathophysiology in ASD.

EEG studies on low-level visual perception found that visually evoked potentials (VEPs) in ASD are less modulated by varying spatial frequencies of stimuli (e.g., in gabor patches or checkerboards) in ASD participants compared to neurotypical controls (“NTs”)^16–19^. An extensive review by Simmons et al.^2^, however, indicates that several EEG studies did not find reliable effects.

### Neural variability

Neurophysiological and imaging studies (e.g., EEG, fMRI) about sensory processing have to deal with low signal-to-noise ratios. One typical way to circumvent this problem is to present the sensory stimuli repeatedly and to average the related sensory signals over repetitions. EEG studies on visual perception, for example, calculate event-related potentials (“ERPs”) or more specifically visually evoked potentials (VEPs, e.g.,^20,21^).

The underlying assumption, is that with each repetition the stimulus is processed in the same way, with the same timing and thus evoke the same physiological signals. Typically, the ERP amplitudes or the average power in certain frequency bands are then analyzed and the variability of the EEG response across repetitions is ignored (analogous approaches are used for fMRI analysis). However, a closer look indicates that neural variability is not necessarily an irrelevant or even distracting by-product of sensory processing. Neural variability can have rather important functional roles for the brain, as has already been discussed 50 years ago^22,23^. Studies on stochastic resonance indicate that the right amount of noise in the neural processing chain can elevate signals evoked by a sub-threshold stimulus, thereby increasing the probability to elicit enough spikes to make them consciously detectable^24,25^. This in turn can adapt detection thresholds in the system, thus increasing perceptual sensitivity under certain circumstances (see^26^ for a review). Furthermore, neuronal networks that learn under noisy conditions have shown to be more robust to disruption, which benefits learning and adaptation^27^.

### Altered neural variability in ASD?

Important for the present study are findings of larger neural variability in ASD participants compared to NTs^19,28–32^. The authors postulated that ASD participants show larger fluctuations in the neural response to repeated sensory (visual, auditory or somatosensory) stimulation. Butler et al.^33^ labeled this approach as the “neural unreliability thesis of autism” and tested it in an EEG study with ASD children and matched controls. No evidence for this neural unreliability thesis was found.

Notably, the analytical protocols used in the aforementioned studies on neural variability in ASD differed significantly. Milne^28^ calculated coefficients of variation (CV) by normalizing the variability of the P1 ERP component by its median amplitude. The rationale behind this is that EEG variability scales with mere EEG amplitudes and therefore needs to be corrected in order to reduce inter-individual variability. Weinger et al.^31^ followed a different approach, both in terms of the experimental paradigm and the analytical protocol. They induced a steady-state EEG response by presenting a stimulus with a high temporal frequency. Signal-to-noise ratio (SNR) was then calculated by dividing the EEG’s frequency band power at stimulation frequency (regarded as the signal) by the surrounding frequency bins (regarded as an estimate of noise), which yields a measure of how precisely the stimulation frequency is mapped to the EEG. While this measure may be related to neural variability similarly as described by Milne^28^, conceptually it captures a different aspect of variability. Butler et al.^33^ used the inter-trial phase coherence (ITPC) to measure neural variability. This is again a different view on variability since it isolates variability in the phase of signals from variability in amplitudes.

It is so far unclear, whether the neural unreliability in ASD, as reported in the above cited studies, is always present or whether it only affects neural responses to sensory events, whether it is present in the entire brain or restricted to local areas and/or certain processing steps, whether it increases (or decreases) over the course of an experiment (e.g. due to fatigue effects) or whether there is a constant feature over time. The pattern of mixed and partially contradictory findings in the literature concerning neural variability in ASD may thus be explained by different experimental contexts and/or by different measurement points in space and/or time.

The present study is based on a recent EEG experiment executed in our lab^18^, where checkerboard stimuli were presented to a group of AS participants and a group of matched NT participants. We have already published the results from a classical ERP analysis of these data, where we found smaller amplitudes of an early visual ERP signature^18^ in AS compared to NTs. During the ERP analysis we noticed an interesting tendency for smaller variability of this ERP signature in the AS group than in the NT group (see Fig. 4 in^18^), which is opposite to the findings supporting the neural unreliability thesis, as introduced above.

Inspired by the neural unreliability thesis, we focused in the present study on this previously ignored variability pattern in the data from Kornmeier et al.^18^ and tried a more extensive analytical look on variability features. We defined a number of variability measures and tested for their differences between AS participants and NTs at different time scales.

In our study from 2014^18^ we focused Asperger Syndrome (AS), which is a subtype of ASD according to the 10th edition of the *International Statistical Classification of Diseases and Related Health Problems* (ICD-10,^1^, 1992) definition, incorporating “restricted, stereotyped, repetitive repertoire of interests and activities”, in addition to the ASD-typical behaviors. Importantly, AS is not marked by deficiencies in cognitive and language development. The fifth *Diagnostic and Statistical Manual of Mental Disorders* (DSM-5,^5^, 2013 and ICD-11 (^34^,2019) have abandoned the distinction between the three subtypes of autism in ICD-10 (early childhood autism, atypical autism, Asperger’s syndrome). However, Asperger’s syndrome according to ICD-10 is still part of ASD according to ICD-11 and DSM-5. Therefore, studying Asperger’s syndrome according to ICD-10 definition renders a study group most likely more homogenous than studying ASD according to ICD-11.

The topic of variability effects in neural processing in psychiatric patients is a relatively recent one. Studies in general and particularly EEG studies are thus rare so far. This conversely means that we currently have little knowledge about potentially interesting spatial and temporal regions of brain processing. Due to the high dimensionality of the EEG data we are confronted with a large search space. In the current study we thus adopted an exploratory analysis approach including heavy multiple testing. As a consequence of this strategy, any attempt to deal with the resulting problem of alpha error inflation, e.g., by the application of a Bonferroni correction, would require extremely small p-values. We decided to dispense any type of multiple testing correction, instead for all statistical tests applied an alpha of 0.05 and report the calculated p-values together with effect sizes. The reason for this decision was that we regard it at the current stage of this novel research area as more important to collect possibly meaningful patterns than to prove their reliability. Ultimately, reliability issues of statistical results will anyway only be answered by accumulating evidence from follow-up replication studies, in the best case from multiple labs.

In summary, we aim to clarify whether an objective measure of altered neural variability in ASD can be documented in EEG visually evoked potential data. Precisely, we hypothesize altered neural variability at occipital electrodes in the ASD group compared to NTs.

## Results

We compared the number of available trials per condition and group to rule out possibly confounding effects of sample sizes. The number of available trials per participant and stimulus was, however, not significantly different between groups ($F(1, 68)=0.03, p=0.872, \eta ^{2}=3.85 \cdot 10^{-4}$). Bayes factor revealed that there is evidence towards H0 (no difference in trials, $BF_{10}=0.249$).

### Questionnaire scores

AS participants scored AQs between 34 and 50 ($median=44$) and EQs between 18 and 41 ($median=30$). Controls scored substantially lower AQs between 8 and 28 ($median=13.5$) and higher EQs between 36 and 73 ($median=57$).

### Inter-trial variability (ITV)

**Figure 1:**
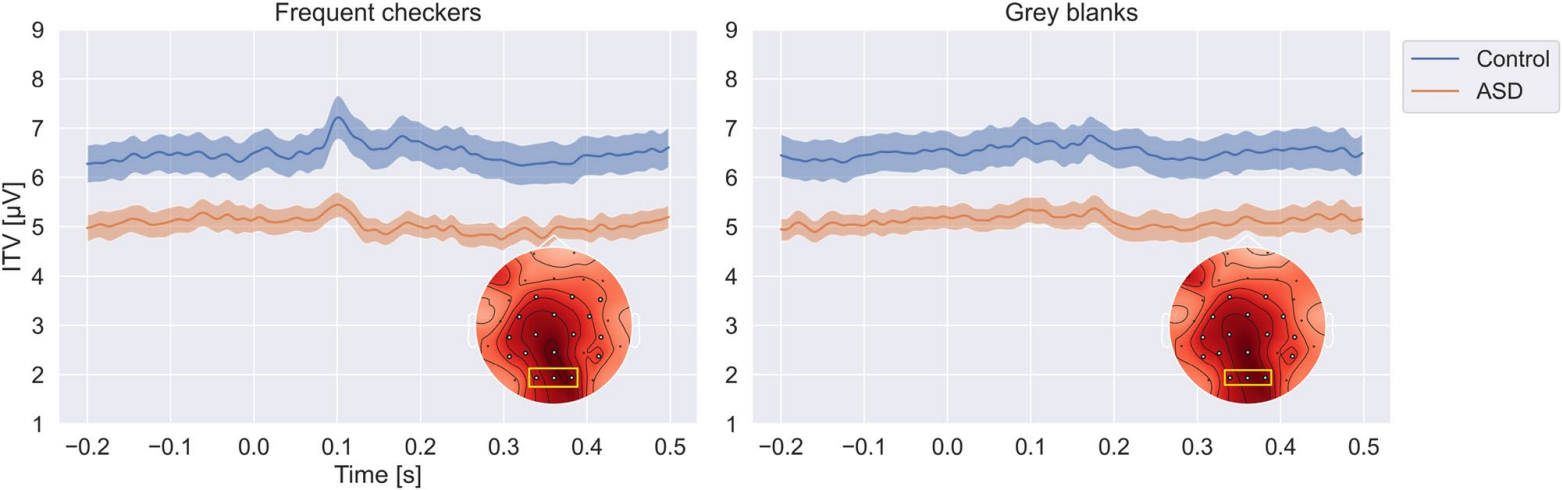
Inter-trial variability between groups. Blue traces show mean ITV of the control group, red shows the mean ITV of the ASD group averaged across the ROI electrodes (O1, Oz, O2; yellow rectangles in the topographic plots). Error shadings indicate group SEM. Left: Frequent checkers. Right: gray blanks. Topographic plots show the channel-wise ITV averaged over the time dimension and contrasted between the groups (NT group—ASD group). Highlighted (white) electrodes indicate a significant difference at criterion $p<0.05$ between groups using an independent two-sided t-test. Note, that ITV is lower in the ASD group regardless of stimulus category (checkerboard or gray screen).

The inter-trial variability (ITV) was calculated for the frequent checkerboards (FC) and gray blanks (GB) time-resolved (i.e. for each time point within the checkerboard and gray screen presentation time windows) for the spatial ROI (the occipital electrodes, see Fig. 1, red and blue traces). In an additional exploratory step we calculated ITV separately for each electrode but averaged the ITV values over time. The interpolated results of this analysis are reflected in the topographic plot (Fig. 1).

We found lower ITV for the ASD group for both the checkerboard stimuli and the gray blanks. Statistical evaluation of the ITV effect was carried out using a two-factor ANOVA on ITVs averaged across the time dimension. This revealed a highly significant difference of ITV between groups ($F(1, 206)=82.35, p=4.38 \cdot 10^{-12}, \eta ^{2}=0.21$). No significant difference was found for the factor stimulus ($F(1, 206)=0.11, p=0.745, \eta ^{2}=0.0004$) and no significant interactions were indicated. In summary, the ITV differs strongly between groups, but this difference seems to be independent of the specific visual stimulation, i.e. whether the checkerboards or the gray screen without any checkerboards were presented. Our additional spatial analysis further indicated very similar spatial distributions of the ITV across checkerboard stimuli and gray screen stimulation (see Fig. 1). The ITV difference between groups showed a broad distribution from central to occipital electrodes, perhaps indicating that the reduced ITV in ASD compared to controls was not restricted to lower-level visual processing areas.

### Evolving inter-trial variability (ETV)

**Figure 2:**
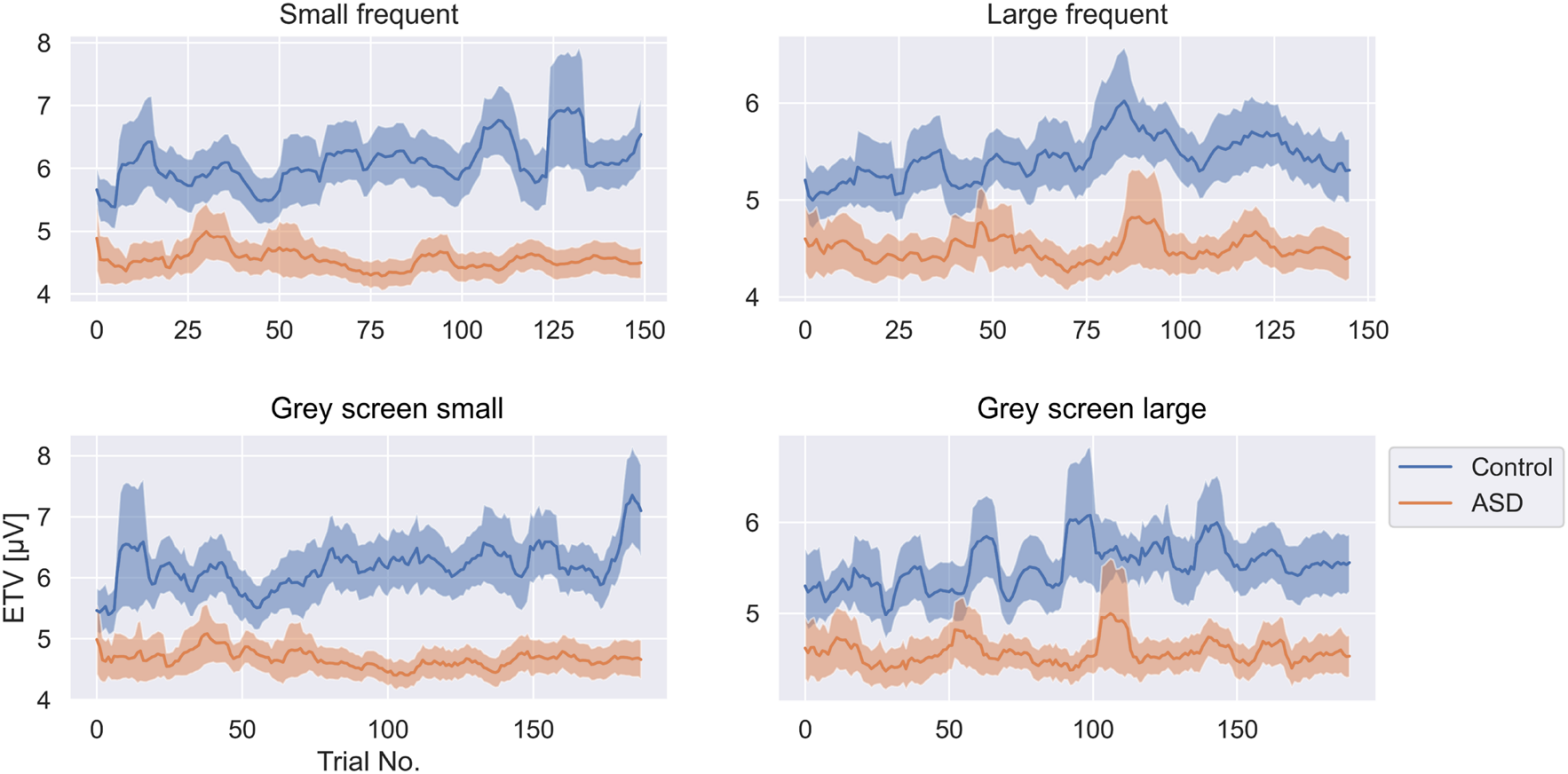
Evolving inter-trial variability. Blue traces show mean ETV of the control group, red traces show the mean ETV of the ASD group. Error shadings indicate group SEM. The ordinate shows the ordered trial number in the experiment. ETV is shown for small frequent and larger frequent checkers, as well as for the gray screens shown in the respective conditions. The bottom left graph depicts the ETV during the gray screen presentations in the small frequent/ large rare checkerboard condition. The bottom right graph shows the ETV during the gray screens in the large frequent/small rare checkerboard condition.

The evolving inter-trial variability (ETV) is a sliding window calculation of ITV spanning over a predefined, ordered subset of trials. It maps fluctuations in ITV over the 5 min of the checkerboard experiment. With this analysis we tested whether the observed ITV difference between groups is present during the whole experiment or whether it is present only during a certain time window within the 5 min duration of an experimental condition, e.g., only during the first or second half of it (see Fig. 2). The lower variability in the ASD group compared to controls was extended over the whole 5 min. In an additional analysis step we focused on the dynamics of the identified ETV effect. For this, we normalized the individual ETV values by dividing them by the respective mean (across time) and calculated several parameters that characterize the ETV dynamics: Slope, ETV variability and detrended ETV-variability.

#### ETV slope

The ETV slope indicates whether ITV tends to increase, decrease or remain stable over the course of the 5-min experiment. The slope of the ETV was calculated for each participant and condition. A two factor ANOVA was calculated to test the differences between groups and stimuli. The ANOVA indicated a significant difference between groups ($F(1, 132)=7.74, p=0.006, \eta ^{2}=0.055$) but no significant differences between stimuli ($F(1, 132)=0.76, p=0.517, \eta ^{2}=0.017$). The ETV slope was significantly larger than 0 for the control group as revealed in a one-sample T-test ($T(75)=5.03, p=4.00 \cdot 10^{-6}, d=0.63, BF_{10}=3929.75$), indicating increasing EEG variability over time. The ETV slopes in ASD were not significantly larger than 0 ($T(75)=0.37, p=0.72, d=0.04, BF_{10}=0.14$) and Bayes factor indicates that there no evidence for a non-zero slope in the ASD group.

#### ETV variability

Closer inspection of Fig. 2 indicated much more dynamics over time in the ETV trace of the control group compared to the ASD group. We calculated the standard deviation (over time) of the ETV as an index of ITV-stability over time. A one-way ANOVA revealed a significant difference in the ETV-variability between the ASD and control group of medium effect size ($F(1, 132)=5.44, p=2.12 \cdot 10^{-2}, \eta ^{2}=0.04$).

#### Detrended ETV-variability

One possible source of the observed ETV variability effect could be driven by the drift of the ITV as reflected in the above reported ETV slope difference. We thus detrended the ETV prior to calculating the standard deviation, leaving only variability that is not related to a global offset in variability or drifts over the experiment. One-way ANOVA revealed even slightly stronger differences between groups ($F(1, 132)=6.16, p=1.43 \cdot 10^{-2}, \eta ^{2}=0.04$) after detrending, which confirms that ITV is not only smaller but also more stable in the ASD group compared to controls.

In summary, the ETV analysis has revealed that ITV trajectories over the 5-minute-course of our experiment are permanently smaller in our AS group compared to the NT group. Moreover, we found that the ITV increases over the course of the experiment in NT, which is not the case in ASD. Furthermore, the detrended fluctuations of the ETV are larger in the control group and these fluctuations increased with the progression of the experiment while they remained small and stable in the ASD group.

### Mechanisms of variability

**Figure 3:**
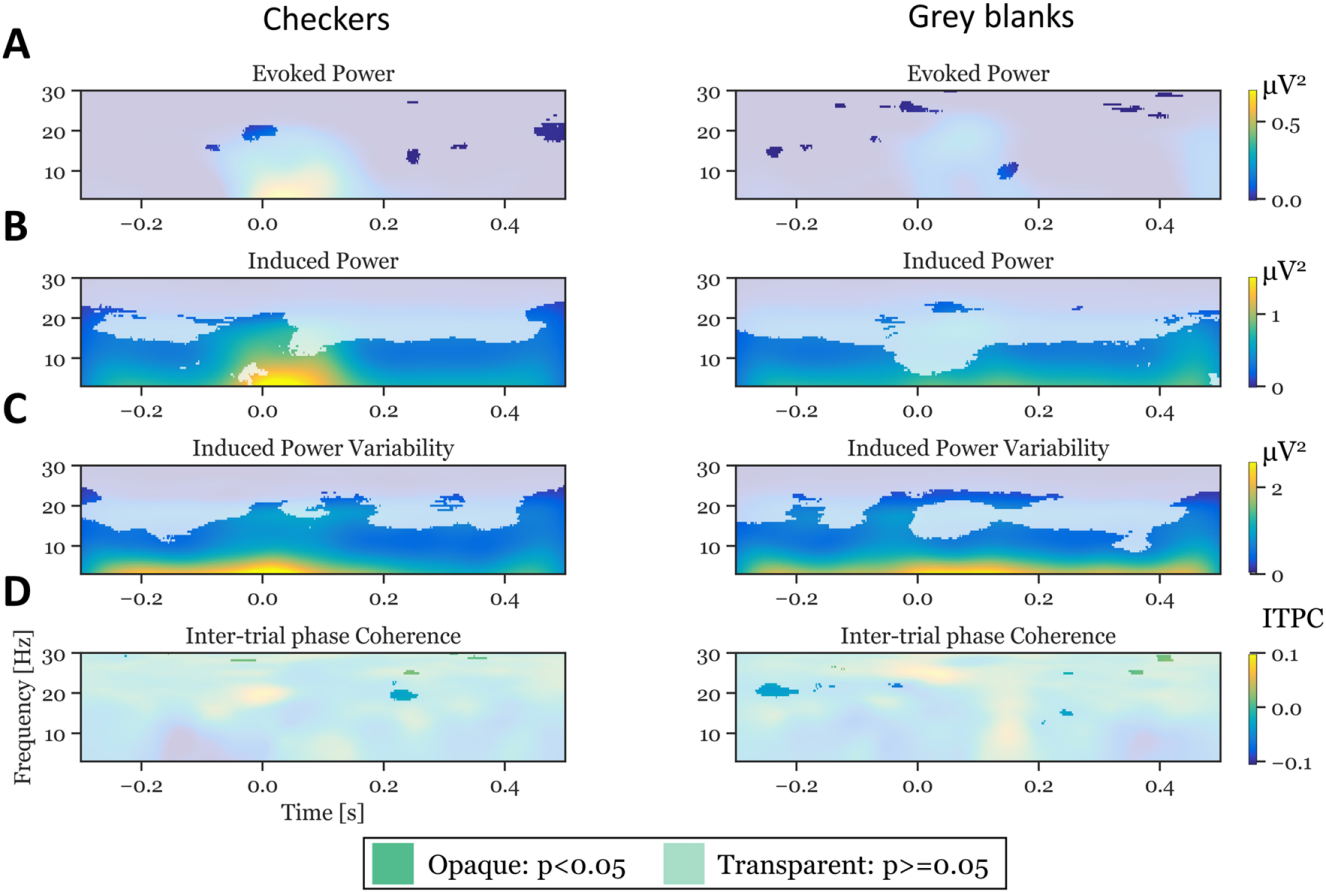
Sources of EEG variability. Column show results for the different stimulus categories checkerboards and gray blanks. Rows show different aspects of time-frequency power and variability. Evoked Power: Time-frequency analysis of ERPs. Induced Power: Time-frequency analysis of induced (i.e., single-trial based) responses. Induced Power Variability: The standard deviation of power values across trials from single-trial time-frequency analyses. Inter-trial phase coherence: An index of phase coherence between trials. Each panel depicts the group difference control group—ASD group. Opaque colors indicate a significant difference at the given time-frequency bin using a Wilcoxon test with an alpha criterion of 0.05. We find that the most consistent differences between groups are reflected in induced responses and their variability, but not in evoked responses or in phase coherence.

In an effort to better understand the mechanism of these variability differences we calculated time-frequency analysis for the ERP (“evoked activity”) and on the single trial level (“induced activity”). We found no significant group differences for evoked activity, indicating that the central tendency of evoked neural responses is same in ASD and controls (see Fig. 3A). We found significant and temporally extended differences between groups in the induced activity (Fig. 3B) in the lower frequency range ($\approx 3-15 Hz$). This variability between single trials is also depicted in the inter-trial power variability (“ITPV”, Fig. 3C), which was significantly increased in the control group compared to ASD over the whole stimulus presentation time window. Analysis of inter-trial phase coherence (“ITPC”) revealed no systematic difference between groups.

### Classification using variability metrics

In order to estimate the predictive power of the variability-related metrics we applied an SVM to classify subjects based on their variability measures (Table 1). ITV was the best predictor of group as indicated by a cross-validated accuracy of $74.4\%$, whereas the lowest predictive power was yielded by the ETV-variability metric. The combination of all metrics allows for a cross-validated accuracy of $68.6\%$, which was considerably lower than classifying based on ITV alone. This discrepancy may be due to the small number of participants per group, which poses a major limitation of the power of classifiers in general.

**Table 1:** Classification accuracy.

Metric	ITV	ETV-slope	ETV-variability	ETV-variability (detrended)	Combined
Accuracy	74.3%	54.2%	51.4%	54.3%	68.6%

The cross-validated classification accuracy indicating how well the ASD group can be separated from the control group using each individual variable and all variables combined. The best accuracy was achieved using ITV, reaching a cross-validated accuracy of 74.3%.

### Correlations of the variability measures with AQ and EQ

**Table 2:** Correlation of scores and metrics.

Score	ITV	ETV-slope	ETV-variability	ETV-variability (detrended)	Combined
AQ	− 0.33*	− 0.22	− 0.13	− 0.14	0.30
EQ	0.47**	− 0.13	0.28	0.28	− 0.25

The Pearson correlation coefficients between variability-related metrics and the AQ & EQ questionnaire scores. The correlation between questionnaire scores and all variables combined were calculated using the hyperplane distance of the SVM (see “Methods” section for explanation). Statistically significant correlations are shown in bold.

*$$p<0.05$$; **$$p<0.01$$.

If the calculated variability metrics are associated with ASD they should be correlated with severity of symptoms. We used the AQ and EQ scores to calculate correlations between symptom severity and various variability metrics (Table 2). Furthermore, we estimated the 95% confidence interval (CI) with the bootstrap method. We found that only ITV was significantly correlated with AQ ($r=-0.33, 95\% CI [-0.40,-0.31]$) and EQ scores ($r=0.47, 95\% CI [0.42,0.54]$).

Taking the absolute values of these correlation coefficients (taking into account the expectation of opposing signs of the correlation coefficients for AQ and EQ) the CI of the two correlations do not overlap. Therefore, we conclude that the correlation between ITV and EQ is significantly larger. Other metrics or a combination thereof did not significantly correlate with AQ or EQ scores.

## Discussion

In this study we addressed the research question of a putative altered variability in basic visual information processing in ASD, using novel analyses of electrophysiological (EEG) data. As a first step, we analyzed the inter-trial variability (ITV) of visually evoked EEG responses across the time course of the trial and found lower variability in ASD throughout the time window.

In a second analysis step we investigated how the ITV effect varies on a larger timescale, across the 5 min duration of the checkerboard experiment. The effect was present for the whole 5 min but increased in the second half of the experiment. The observed variability effects could be restricted to an induced power together with induced power variability in the lower EEG frequency band (3–15 Hz). Interestingly, we found significant correlations between our measures of neural variability and symptom severity as measured with AQ and EQ scores.

The raw EEG we recorded reflects a mixture of neural processes related to stimulus processing and background activity which is unrelated to stimulus processing. The stimulus-specific EEG signatures can be further subdivided into EEG signatures that are precisely time-locked to stimulus onset. The evoked EEG power reflects those processes. Some stimulus-related neural processes, on the other hand, may vary in latency to some degree with respect to stimulus onset. The induced EEG power reflects the sum the time-locked and the non-time-locked or less-time-locked processes.

Our two measures ETV and ITV do not differ between these types of EEG components. The EEG frequency domain, however, is more informative in this respect.

Postulating that EEG variability scales with EEG amplitudes, one would expect that less EEG variability comes with smaller EEG amplitudes. If the observed smaller amplitude variability in ASD reflects differences in perceptual processes time-locked to stimulus onset, this should be reflected in smaller evoked power. Our analyses reveal no difference in the evoked EEG power (see Fig. 3A).

However, even if the evoked power is of comparable magnitude between groups, it may still be possible that single trial EEG amplitudes are in one group larger than in the other group, but a concurrently larger temporal jitter in processing steps underlying checkerboard perception across EEG trials covers this in the time-locked analysis. In this case we would expect significantly smaller induced power (which includes also the power of the not time-locked oscillatory activity) together with smaller ITPC, as a measure of temporal jitter, in the ASD group compared to the control group. Although we found smaller induced power in the ASD group compared to the controls across the whole 500-ms time window for both checkerboard stimuli and gray screen trials (Fig. 3B), no corresponding difference in the ITPC could be observed (Fig. 3D). This is in concordance with ^13^, who also did not find any difference in ITPC in their cohort, whereas ^42^ did report an ITPC effect. These findings indicate that the variability effects, we found, may not be related to the processing of the visual stimulus.

In contrast to the repetitive neural activity related to the repetitive stimulus, the background activity can be regarded as non-repetitive over the course of the EEG measurement. As a consequence, the phases of the contributing oscillations can be expected to have broad distributions over trials. The ITPC measure includes both the stimulus-related processing and the unrelated neural background activity. The absence of an ITPC difference between groups indicates the absence of phase differences in this background oscillatory activity. However, group differences in the power of this background activity may explain the observed differences in both the induced power and in the induced power variability. We thus conclude that the smaller ITV and ETV in the ASD group compared to the control group reflects a generally reduced neural background activity.

This interpretation is further supported by three additional observations. First, the variability effect extends across the whole stimulus presentation time window (the smaller time scale) and also across the whole 5 min duration of the checkerboard experiment (the larger time scale). It can thus not be narrowed down to specific processing steps taking place at specific time instants during stimulus processing. Second, the variability effect is also present in the gray screen interval between checkerboard presentations. Third, in the Appendix A (see Supplementary Material) we present a separate analysis of the amplitude variability of the visual P100 ERP component evoked by the checkerboard stimuli. Following the analysis steps from Milne^28^ we took the coefficient of variance as dependent variable, which represents a variability measure normalized by the P100 amplitude. We found no difference between participant groups, further indicating that the stimulus related processing is unaffected by the observed effects.

It might be the case that the altered background activity is related to alterations of overall different long-distance and local-network brain connectivity^35^ or the altered excitation-inhibition-equilibrium in ASD^36^. To test this, future research should combine such functional investigations for example with MRI- or source-based measurements of neuronal-network connectivity^35,37–39^ or cerebral neurochemistry^40^.

The ITV and ETV effects in the present study encompass the whole 500-ms time window of checkerboard stimulation and are even present if only a gray screen is presented. The identified spatial pattern of the effects (scalp maps in Fig. 1) is universal across processing of the checkerboards and processing of the gray screen. This generality across time and stimulus categories may be interpreted as evidence for trait effects of the AS participants. However, if this was the case one should expect confirmatory findings in the literature. Trial-by-trial variability in neuronal signals in ASD has already been investigated and discussed in a few studies in the past, however with heterogeneous results^19,28–30,32,33,41–44^.

As already discussed in “Introduction” section, the analysis of the data differed strongly between studies. Milne^28^, e.g., decided to normalize variability by amplitude using the coefficient of variation (CV). This type of analysis is not suitable in the present case, because our temporally extended analysis time windows include zero-crossings. Further, on the background of the present analysis, amplitude normalization would not only reduce the potentially distracting effects of inter-individual variability. It would also affect the effect as such, as discussed in more detail in “Normalizing brain activity” section.

There are several important factors that need to be taken into account for the interpretation of certain patterns as state effects or trait effects. One is of course the type of data under consideration. Some studies used EEG, others used fMRI, and it is controversially discussed to what degree variability in fMRI reflects variability in neural processing (e.g.,^33^). Further, it is so far unclear to what degree neural variability in response to visual processing in ASD is different between various ASD endophenotypes and how much it changes over the ontogenetic development. The latter is particularly relevant because most of the above cited studies measured children (e.g.,^28,29,33,45^) whereas the present study investigated adults.

In summary, based on the available studies it is yet not possible to explain the overall controversy between study results. Important factors for the present study may be the choice of the visual stimuli, e.g., the specific sizes of the checkerboards that may provide enough details to attract the perceptual system of ASD observers. The easy structure of our checkerboard oddball experiment (only two conditions, only two stimulus sizes and a gray screen, fixed block length, etc.) may have made the “nature” of upcoming stimulus sequences highly predictable and may have induced a specific experimental state in our AS participants with overall lower neural background activity for the 5-min-period of this specific experiment. The present pattern of results may be not replicable in children with ASD, with longer or shorter experiments or experiments with other—potentially less detailed or less predictable—visual stimuli or with stimuli from other modalities. More studies on EEG variability with varying stimuli need to be executed in order to get a clearer picture and to resolve existing discrepancies. Possibly, this may lead to the discovery of variability-related subgroups.

One explanation for the inconsistent findings of altered neural variability in ASD could be that there is no underlying effect. Positive findings in either direction could be considered sampling errors that arise due to low sample sizes. The observation that our variability effects are not restricted to a single time point but instead show a consistent and stable extension over time makes an alpha error relatively improbable. However, we are aware that further confirmatory studies are required, preferably with high sample sizes.

The present finding of lower EEG variability in ASD participants compared to matched controls is only informative if it covaries with typical ASD symptoms. We thus calculated correlations between our measures of variability and the AQ and EQ scores and found correlations in the range of $0.4< r < 0.5$ (absolute values) with maximal correlation between AQ/EQ and ITV. Particularly, we found stronger correlations of the EEG variability with the EQ than with the AQ scores. As Baron-Cohen and Wheelwright^46^ pointed out in their article on the EQ, empathy can be best defined from a cognitive and an affective perspective. From the cognitive approach, empathy requires analytical thinking, understanding the other’s feelings (“theory of mind”, e.g.,^47^). The fact, that the correlation between EQ and ITV is stronger than that of AQ points to a specific link between the neuronal mechanisms underlying ITV and empathy generation. This relationship has to be clarified in future research.

We also found maximal predictive power of 74.3% with ITV. Of course, this is only a rough estimate, given the relatively small sample size of the present study (see e.g.,^48^ for a discussion of sample size and classifier accuracy). Future studies including larger sample sizes may refine this measure.

Two caveats of the present results are, that they are based on heavy multiple testing and without correction and that sample sizes are relatively low. Any interpretation of the current results thus needs to be regarded under this constraint.

In the present study we found robust and extended (over time, stimulus type and electrode locations) differences in neural variability between AS participants and controls. Particularly, our findings indicate that smaller neural variability in AS participants is linked to neural background activity during the processing of repetitive sensory information. While the present study is clearly explorative in nature and needs replication, the robust pattern of results is a strong indication that the so far widely neglected topic of neural variability needs more investigation, particularly in ASD research.

## Methods

### Participants

EEG data of 21 AS participants and 17 NTs from a study described in Kornmeier et al.^18^ was re-analyzed. NT participants were selected to match the AS participants in age (± 3 years) and gender. All participants had German school education comparable to junior high school or high school. Due to technical reasons only 19 AS participants (age range: 24–59, age mean ± SD: 41.3 ± 10.7; 6 females) and 16 NTs (age range: 22–57, age mean ± SD: 38.8 ± 11.5, 6 females) were used for the analysis. Two participants were excluded due to missing trigger signals and one participant was excluded due to numerous artifacts. The same participants had been excluded in the first publication of the data^18^.

All participants completed the Autism-Spectrum Quotient Test “AQ”^49^ and the Empathy Quotient “EQ”^46^. They had a normal visual acuity as measured with the Freiburg Visual Acuity Test (FrACT,^50^). All participants gave their informed written consent. The study was performed in accordance with the ethical standards laid down in the Declaration of Helsinki^51^ and was approved by the ethics board of the Albert-Ludwigs-Universität Freiburg, Germany. For a detailed description of clinical diagnostics please refer to the earlier publication^18^ on the same cohort.

### Visual stimuli

The stimuli consisted of fine and coarse checkerboards with check sizes of $0.6^{\circ }$ and $1.2^{\circ }$ visual angle (corresponding to 1.67 and 0.8 cycles per degree; cpd) and a gray screen following each checkerboard presentation. Checkerboards and gray screen extended over a field of $13.25^{\circ }$ (width) $\times 14.25^{\circ }$ (height) visual angle. Luminance of the white and black checks was $220 cd/m^2$ and $1.55 cd/m^2$. Luminance of the gray screen was $110 cd/m^2$.

### Experimental procedure

**Figure 4:**
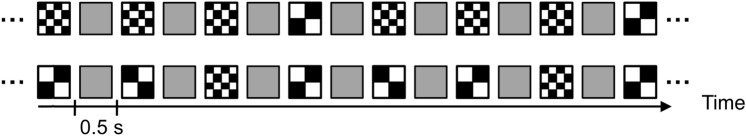
Checkerboard oddball paradigm. Rare checkerboards with coarse check sizes are randomly interspersed with frequent checkerboards with fine check sizes (top row, condition FF) and vice versa (bottom-row, condition CF). Participants were instructed to count the occurrences of the rare checkerboards within an experimental block. Each checkerboard (coarse or fine) was presented for 500 ms and alternated with a gray screen interval, which was also present for 500 ms.

In a balanced experimental paradigm checkerboards with fine/coarse checks were presented as frequent/rare stimuli in the one experimental condition and vice versa in the other condition (coarse checkerboards frequent, see Fig. 4). Each checkerboard stimulus was presented for 500 ms and followed by a gray screen. The rare checkerboards appeared with a probability of $p=0.2$ in a pseudo-random order. Participants were instructed to fixate a centrally presented gray cross and to count the occurrence of rare stimuli.

In summary the current experiment differs between

- two types of stimuli: checkerboards with (1) fine checks and (2) coarse checks
- two experimental conditions: (1) fine checks frequent & coarse checks rare and (2) coarse checks frequent & fine checks rare

We focused our analysis on two different data sets: (1) EEG data to frequent checkerboards (FC data sets), (2) EEG data to the gray screens (GB data sets).

### EEG data acquisition

EEG was recorded with a Brain Vision ActiCHamp amplifier and 32 electrodes of the 10–20 system. Data was sampled at a frequency of 500 Hz using an online high-pass at 0.01 Hz and low-pass at 120 Hz.

### EEG data pre-processing

All data processing was accomplished using Python 3.7.4 and the MNE package (v 20,^52^). The EEG was re-referenced to averaged mastoid electrodes (TP9, TP10) after the recording. An offline band-pass filter was applied between 1 and 30 Hz pass-band edge. Independent component analysis (ICA,^53^) was calculated per subject and components that captured eye artifacts were automatically selected and removed from the data using standard MNE procedures. On average, 1.05 eye components were identified and subsequently removed.

The cleaned data was then cut into trials based on stimulus onset and categorized into stimulus identity (coarse vs. fine frequent checkerboards). Additionally, the gray screens in between the checkerboard presentations were extracted.

Subsequently, bad channels were identified using Random Sample Consensus (RANSAC,^54^) implemented in the Python package *autoreject*^55^. In short, the RANSAC method labels those channels as bad that have low correlation (r< 0.75) with their neighbors over extended time periods ($\approx 40\%$ of the recording). The RANSAC method found on average 1.7 bad channels per participant.

In the current study we focused our analysis on EEG variability across stimulus repetitions at different time scales. For this we did not apply a baseline correction as is a recommended procedure when inter-trial variability is of interest (see e.g.,^56^). Trials with amplitudes exceeding 100 $\mu V$ were marked as missing values and thus did not enter the variability calculations.

### EEG data analysis

The present EEG data had already been used for a classical ERP analysis by Kornmeier et al.^18^. They found larger ERP checksize effects in AS participants than in NTs over occipital electrodes. We therefore defined a region of interest of the electrodes O1, Oz and O2 in order to capture visual responses from the primary visual cortices.

#### Inter-trial variability (“ITV”)

**Figure 5:**
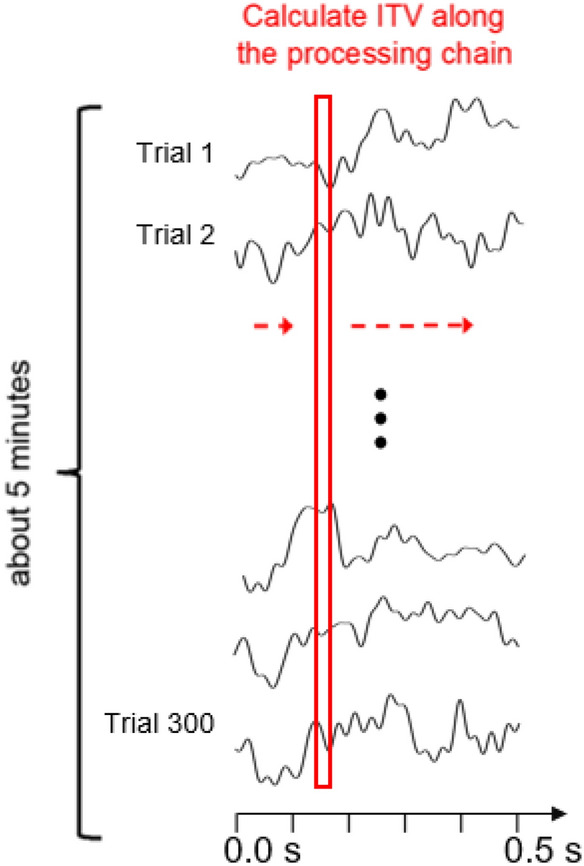
ITV calculation. The inter-trial variability (ITV) corresponds to the EEG standard deviation at a certain time point during stimulus presentation across repeated stimulus presentations. ITV is calculated for each time point during the stimulus (checkerboard or gray screen) presentation time window of 500 ms, as indicated by the red rectangle.

For a given participant and condition (FC or GB), the ITV was computed on the EEG data ${\textbf {M}} \in {\mathbb {R}}^{trials \times channels \times time}$ for each electrode j from the spatial ROI and time point k as the standard deviation across P trials:

1 $$\begin{aligned} ITV_{(j, k)} = \sqrt{ \frac{1}{P} \sum _{p=1}^{P} (M_{p, j, k} - \bar{M}_{j, k})^{2} } \end{aligned}$$

where $\bar{M}$ is the EEG averaged over trials (i.e. ERP). This value corresponds to the point- and electrode-wise standard deviation over stimulus repetitions (trials). A visual explanation is given in Fig. 5.

#### Evolving inter-trial variability (“ETV”)

**Figure 6:**
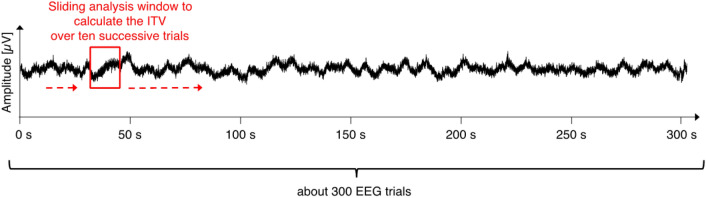
ETV calculation. The black fluctuating trace represents the raw EEG trace from the occipital electrode of one example participant observing repeated checkerboards and gray screens over a time course of 5 min. The evolving inter-trial variability (ETV) corresponds to calculation of the average of the ITVs over time within a sliding (indicated by the red dashed arrows) time window encompassing ten successive EEG trials (indicated by the red rectangle). This calculation results in the evolution of the average ITV over the whole experiment (i.e., 5 min or 300 EEG trials), thus reflecting EEG variability at a coarser timescale, compared to ITV.

Additionally, we were interested whether the neural variability changes (or stays stable) over a larger timescale across the whole experiment. We thus focused on the evolution of the ITV over the 5 min duration of the checkerboard experiment (see Fig. 6 for a visual explanation). We calculated the *evolving* ITV (“ETV”) in a sliding-window-procedure over the chronologically ordered trials. The window width to calculate the ITV extended over 10 trials, i.e., the standard deviation was calculated for the first 10 trials per channel and time point. In a second step the calculated standard deviation values were averaged across the 500 trial data points, resulting in one ITV value. Then the window slides one trial further and the second ITV value was calculated for the 2nd to the 11th trial and so forth. This calculation was performed separately for each of the ROI electrodes and for each participant and stimulus.

#### ITPC and ITPV

In addition to the ITV analyzes, we distinguish two theoretically independent mechanisms that could drive ITV: (1) Differences in inter-trial phase coherence (ITPC) and inter-trial power variability (ITPV). The basic idea of these measures is the following: The EEG raw traces can be regarded as a superposition of oscillatory activity of a number of neural generators. Oscillatory activity in different frequencies can be calculated by frequency analysis tools providing power values and respective phase values. Phase and power per frequency can then be compared across EEG trials. Phase consistency, measured by ITPC, evaluates the similarity of phase angles across trials per frequency band and time frame and yields a phase coherence index between 0 (not aligned) and 1 (exactly aligned). ITPV measures the variability of power responses in the respective frequencies over trials. ITPC is thus a measure of timing accuracy, whereas ITPV reflects how the amplitudes vary across trials; thereby neglecting phase angles. ITPV can be viewed as a more elaborate ITV measure that allows for frequency-specific resolution and incorporates both evoked and ongoing neural oscillations.

### Normalizing brain activity

The EEG is highly variable between participants. While some of this variability can be explained by differences in functional organization of the individual brains, some of it can be explained anatomically by differences in skull thickness, tissue conductivity, gray matter density, etc. These factors have a multiplicative effect on the amplitudes recorded by the EEG, which can be corrected by normalization. The separation of the impacts of these different sources is difficult.

In the work by Milne^28^, variability was normalized to account for these factors, as is common practice. In the present work we were explicitly interested in within-participant variability across repetitive visual stimulation at different time scales (trial-by-trial variability). Normalization could potentially remove a significant portion of the trial-by-trial variability and thus reduce potential between group effects. Instead, we additionally analyzed induced and evoked responses, which is a more sensible approach without loss of information. In order to provide a link between our analyses and those by Milne^28^ we have added an analysis of normalized variability in Appendix Fig. 1

#### Classification analysis

In order to show the discriminatory power of the variability metrics introduced above we trained a Support Vector Machine (SVM) classifier^57^ using all variability scores. The hyperparameters of the SVM were tuned using Bayesian optimization^58^ and the accuracy scores were cross-validated using *Leave-one-out cross-validation*. The relative distance from the data sample to the hyperplane, as well as what side of the hyperplane the sample was located, was computed for each participant. This relative distance is comparable to a confidence score. The distances were correlated with behavioral scores of the AQ and the EQ. The rationale behind this approach is to investigate the discriminatory power of all variability scores and combination of variability scores and to efficiently determine their relation to the AQ and EQ scores.

### Statistical analysis

Statistical analysis was performed using analysis of variance (ANOVA) and independent, two-sided t-tests. As a measure of effect size we calculated Cohen’s d (*d*,^59^). Where parametric tests revealed no significant differences a “Bayesian t-test” was calculated, using the approach described by Rouder et al.^60^, to decide whether there was evidence for the null hypothesis. The Jeffrey–Zellner–Siow prior was used, which is the Cauchy distribution on effect size. A Bayes factor ($BF_{10}$) larger 3 is considered evidence for ${\mathcal {H}}1$., whereas a Bayes factor smaller 0.3 is considered evidence for the null hypothesis (${\mathcal {H}}0$). The Pingouin package for Python^61^ was used for all statistical tests.

### Ethics approval

All participants gave their informed written consent. The study was performed in accordance with the ethical standards laid down in the Declaration of Helsinki^51^ and was approved by the ethics board of the Albert-Ludwigs-Universität Freiburg, Germany.

## Supplementary Information

Supplementary Information.

## Data Availability

The datasets generated during and/or analysed during the current study are available from the corresponding author upon reasonable request.

## Code availability

All code that was used to analyze and visualize the data and to calculate the statistics is available here: https://github.com/LukeTheHecker/asd-variability.

## References

[1] World Health Organization. *The ICD-10 Classification of Mental and Behavioural Disorders: Clinical Descriptions and Diagnostic Guidelines* (1992).

[2] Simmons DR, Robertson AE, McKay LS, Toal E, McAleer P, Pollick FE (2009). Vision in autism spectrum disorders. Vis. Res..

[3] Marco EJ, Hinkley LBN, Hill SS, Nagarajan SS (2011). Sensory processing in autism: A review of neurophysiologic findings. Pediatr. Res..

[4] Robertson CE, Baron-Cohen S (2017). Sensory perception in autism. Nat. Rev. Neurosci..

[5] American Psychiatric Association. *Diagnostic and Statistical Manual of Mental Disorders (DSM-5®)* (American Psychiatric Pub, 2013).

[6] Bogdashina, O. *Sensory Issues in Autism: Different Sensory Experiences Different Perceptual Worlds* (2003).

[7] Allen ML, Chambers A (2011). Implicit and explicit understanding of ambiguous figures by adolescents with autism spectrum disorder. Autism.

[8] Frith, U. *Autism: Explaining the Enigma* (Blackwell, 2003).

[9] Happé F, Frith U (2006). The weak coherence account: Detail-focused cognitive style in autism spectrum disorders. J. Autism Dev. Disord..

[10] Joseph RM, Keehn B, Connolly C, Wolfe JM, Horowitz TS (2009). Why is visual search superior in autism spectrum disorder?. Dev. Sci..

[11] Mitchell P, Ropar D (2004). Visuo-spatial abilities in autism: A review. Infant Child Dev. Int. J. Res. Pract..

[12] Plaisted K, O’Riordan M, Baron-Cohen S (1998). Enhanced visual search for a conjunctive target in autism: A research note. J. Child Psychol. Psychiatry Allied Discipl..

[13] Happé FGE (1996). Studying weak central coherence at low levels: Children with autism do not succumb to visual illusions. A research note. J. Child Psychol. Psychiatry.

[14] Kornmeier, J., Wörner, R., Riedel, A., Tebartz van Elst, L. A different view on the Necker cube—Differences in multistable perception dynamics between Asperger and non-Asperger observers. *PLoS ONE* **12**(12), e0189197 (2017). 10.1371/journal.pone.0189197 PMC5731733 29244813

[15] Tebartz van Elst L, Bach M, Blessing J, Riedel A, Bubl E (2015). Normal visual acuity and electrophysiological contrast gain in adults with high-functioning autism spectrum disorder. Front. Hum. Neurosci..

[16] Boeschoten MA, Kenemans JL, van Engeland H, Kemner C (2007). Abnormal spatial frequency processing in high-functioning children with pervasive developmental disorder (PDD). Clin. Neurophysiol..

[17] Jemel B, Mimeault D, Saint-Amour D, Hosein A, Mottron L (2010). VEP contrast sensitivity responses reveal reduced functional segregation of mid and high filters of visual channels in autism. J. Vis..

[18] Kornmeier, J., Wörner, R., Riedel, A., Bach, M., & Tebartz van Elst, L. A different view on the checkerboard? Alterations in early and late visually evoked EEG potentials in asperger observers. *PLoS ONE* **9**(3), e90993 (2014). 10.1371/journal.pone.0090993. 10.1371/journal.pone.0090993 PMC3954585 24632708

[19] Milne E, Griffiths H, Buckley D, Scope A (2009). Vision in children and adolescents with autistic spectrum disorder: Evidence for reduced convergence. J. Autism Dev. Disord..

[20] Kornmeier J, Bach M (2004). Evidence for early visual processing in perceptual disambiguation of ambiguous figures. J. Vis..

[21] Heinrich SP, Lüth I, Bach M (2015). Event-related potentials allow for optotype-based objective acuity estimation. Investig. Ophthalmol. Vis. Sci..

[22] Callaway E, Halliday RA (1973). Evoked potential variability: Effects of age, amplitude and methods of measurement. Electroencephalogr. Clin. Neurophysiol..

[23] Callaway E, Jones RT, Donchin E (1970). Auditory evoked potential variability in schizophrenia. Electroencephalogr. Clin. Neurophysiol..

[24] Benzi R, Sutera A, Vulpiani A (1981). The mechanism of stochastic resonance. J. Phys. A Math. Gen..

[25] McDonnell MD, Ward LM (2011). The benefits of noise in neural systems: Bridging theory and experiment. Nat. Rev. Neurosci..

[26] Aldo Faisal A, Selen LPJ, Wolpert DM (2008). Noise in the nervous system. Nat. Rev. Neurosci..

[27] Basalyga G, Salinas E (2006). When response variability increases neural network robustness to synaptic noise. Neural Comput..

[28] Milne E (2011). Increased intra-participant variability in children with autistic spectrum disorders: Evidence from single-trial analysis of evoked EEG. Front. Psychol..

[29] Dinstein I, Heeger DJ, Lorenzi L, Minshew NJ, Malach R, Behrmann M (2012). Unreliable evoked responses in autism. Neuron.

[30] Dinstein I, Heeger DJ, Behrmann M (2015). Neural variability: Friend or foe?. Trends Cogn. Sci..

[31] Weinger PM, Zemon V, Soorya L, Gordon J (2014). Low-contrast response deficits and increased neural noise in children with autism spectrum disorder. Neuropsychologia.

[32] Haigh SM, Heeger DJ, Dinstein I, Minshew N, Behrmann M (2015). Cortical variability in the sensory-evoked response in autism. J. Autism Dev. Disord..

[33] Butler JS, Molholm S, Andrade GN, Foxe JJ (2017). An examination of the neural unreliability thesis of autism. Cereb. Cortex.

[34] World Health Organization. *ICD-11: International Classification of Diseases (11th Revision)* (2019).

[35] TebartzvanElst L, Riedel A, Maier S (2016). Autism as a disorder of altered global functional and structural connectivity. Biol. Psychiatry.

[36] Tebartz Van Elst L, Maier S, Fangmeier T, Endres D, Mueller GT, Nickel K, Ebert D, Lange T, Hennig J, Biscaldi M (2014). Disturbed cingulate glutamate metabolism in adults with high-functioning autism spectrum disorder: Evidence in support of the excitatory/inhibitory imbalance hypothesis. Mol. Psychiatry.

[37] Schoffelen JM, Gross J (2009). Source connectivity analysis with MEG and EEG. Human brain mapping.

[38] Hecker, L., Rupprecht, R., Tebartz van Elst, L., & Kornmeier, J. ConvDip: A convolutional neural network for better EEG Source Imaging. *Frontiers in Neuroscience*, **15**, 569918. (2021). 10.3389/fnins.2021.569918 PMC8219905 34177438

[39] Hecker, L., Rupprecht, R., van Elst, L. T., & Kornmeier, J. Long-Short Term Memory Networks for Electric Source Imaging with Distributed Dipole Models. *bioRxiv*. 10.1101/2022.04.13.488148 (2022).

[40] Tebartz van Elst L, Maier S, Fangmeier T, Endres D, Mueller GT, Nickel K, Ebert D, Lange T, Hennig J, Biscaldi M, Riedel A, Perlov E (2014). Disturbed cingulate glutamate metabolism in adults with high-functioning autism spectrum disorder: Evidence in support of the excitatory/inhibitory imbalance hypothesis. Mol. Psychiatry.

[41] Easson AK, McIntosh AR (2019). BOLD signal variability and complexity in children and adolescents with and without autism spectrum disorder. Dev. Cogn. Neurosci..

[42] Kovarski K (2019). Reduced visual evoked potential amplitude in autism spectrum disorder, a variability effect?. Transl. Psychiatry.

[43] Sutherland A, Crewther DP (2010). Magnocellular visual evoked potential delay with high autism spectrum quotient yields a neural mechanism for altered perception. Brain.

[44] Chung S, Son J-W (2020). Visual perception in autism spectrum disorder: A review of neuroimaging studies. J. Korean Acad. Child Adoles. Psychiatry.

[45] Brandwein AB, Foxe JJ, Butler JS, Russo NN, Altschuler TS, Gomes H, Molholm S (2013). The development of multisensory integration in high-functioning autism: High-density electrical mapping and psychophysical measures reveal impairments in the processing of audiovisual inputs. Cereb. Cortex.

[46] Baron-Cohen Simon, Wheelwright Sally (2004). The empathy quotient: An investigation of adults with asperger syndrome or high functioning autism, and normal sex differences. J. Autism Dev. Disord..

[47] Gibbs, R.W., Jr. *Embodiment and Cognitive Science* (Cambridge University Press, 2005).

[48] Flint C, Cearns M, Opel N, Redlich R, Mehler DMA, Emden D, Winter NR, Leenings R, Eickhoff SB, Kircher T, Krug A, Nenadic I, Arolt V, Clark S, Baune BT, Jiang X, Dannlowski U, Hahn T (2021). Systematic misestimation of machine learning performance in neuroimaging studies of depression. Neuropsychopharmacology.

[49] Baron-Cohen S, Wheelwright S, Skinner R, Martin J, Clubley E (2001). The autism-spectrum quotient (AQ): Evidence from asperger syndrome/high-functioning autism, males and females, scientists and mathematicians. J. Autism Dev. Disord..

[50] Bach M (1996). The Freiburg visual acuity test-automatic measurement of visual acuity. Optom. Vis. Sci..

[51] World Medical Association. World Medical Association Declaration of Helsinki: Ethical principles for medical research involving human subjects. *JAMA*, **310**(20), 2191–2194 (2013). 10.1001/jama.2013.281053. 10.1001/jama.2013.281053 24141714

[52] Gramfort A, Luessi M, Larson E, Engemann DA, Strohmeier D, Brodbeck C, Goj R, Jas M, Brooks T, Parkkonen L (2013). MEG and EEG data analysis with MNE-Python. Front. Neurosci..

[53] Vigário R, Sarela J, Jousmiki V, Hamalainen M, Oja E (2000). Independent component approach to the analysis of EEG and MEG recordings. IEEE Trans. Biomed. Eng..

[54] Bigdely-Shamlo N, Mullen T, Kothe C, Kyung-Min S, Robbins KA (2015). The PREP pipeline: Standardized preprocessing for large-scale EEG analysis. Front. Neuroinform..

[55] Jas M, Engemann DA, Bekhti Y, Raimondo F, Gramfort A (2017). Autoreject: Automated artifact rejection for MEG and EEG data. NeuroImage.

[56] Arazi A, Gonen-Yaacovi G, Dinstein I (2017). The magnitude of trial-by-trial neural variability is reproducible over time and across tasks in humans. eNeuro.

[57] Pedregosa F, Varoquaux G, Gramfort A, Michel V, Thirion B, Grisel O, Blondel M, Prettenhofer P, Weiss R, Dubourg V, Vanderplas J, Passos A, Cournapeau D, Brucher M, Perrot M, Duchesnay E (2011). Scikit-learn: Machine learning in python. J. Mach. Learn. Res..

[58] Nogueira, F. *Bayesian Optimization: Open Source Constrained Global Optimization Tool For Python* (2014).

[59] Cohen J (1992). Statistical power analysis. Curr. Direct. Psychol. Sci..

[60] Rouder JN, Speckman PL, Sun D, Morey RD, Iverson G (2009). Bayesian t tests for accepting and rejecting the null hypothesis. Psychonom. Bull. Rev..

[61] Vallat R (2018). Pingouin: Statistics in python. J. Open Source Softw..

