# Supplementary material for: Altered EEG variability on different time scales in participants with autism spectrum disorder: an exploratory study
Source: Sci Rep. 2022 Jul 29;12:13068. doi: 10.1038/s41598-022-17304-x (PMC9338240; doi:10.1038/s41598-022-17304-x)
Supplement: Supplementary file 1 — Supplementary Information. [file 41598_2022_17304_MOESM1_ESM.pdf]

1 Appendix A

Figure 1 shows the coefficients of variation of the P1 responses to frequent checkerboards for the two groups. Methodically, we have exactly replicated the approach described by [28]. Only trials in which frequent checkers were presented have been selected for this analysis. Baseline corrected EEG epochs were used for the following analyses. As a first step, we have selected the channel that showed the highest P1 amplitude from current source density interpolated data. Typically, this procedure selected an occipital or parietal electrode. Next, the peak amplitude within 100-170 ms post-stimulus time interval was found for each trial. The coefficient of variation (CV) of the P1 peak amplitudes was then calculated as the median absolute deviation divided by the median P1 peak amplitude. A t-test for independent samples revealed a small, non-significant, effect ($t = 0.91, p = 0.372, d = 0.31$).

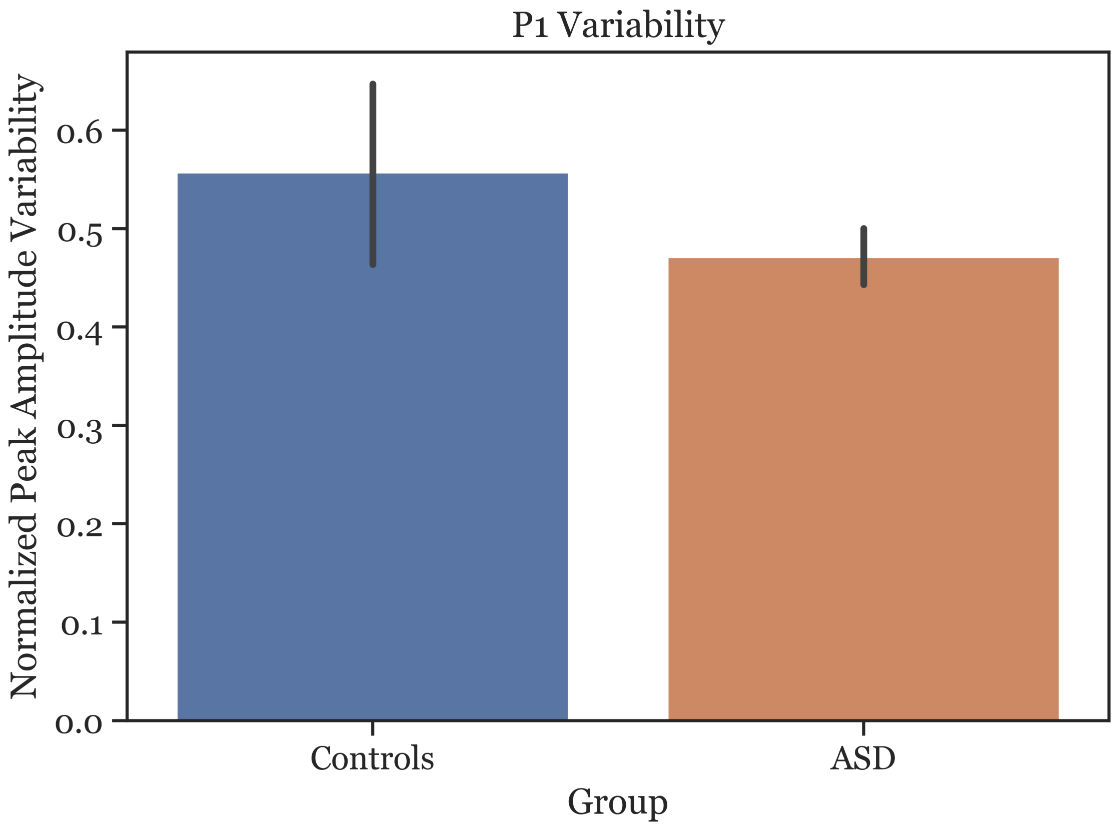

Figure 1: **P1 Variability.** Coefficients of variation (CV) of the P1 responses are shown for neurotypicals (blue bar) and the ASD group (orange bar). CVs were calculated as the median absolute deviation of single-trial P1 peak amplitudes divided by the median peak amplitude. Error bars indicate standard errors of the mean (SEM).
